# Food-Related Illness and Death in the United States Reply to Dr. Hedberg

**DOI:** 10.3201/eid0506.990625

**Published:** 1999

**Authors:** Paul S. Mead, Laurence Slutsker, Patricia M. Griffin, Robert V. Tauxe

**Affiliations:** Centers for Disease Control and Prevention, Atlanta, Georgia, USA


similar to *Campylobacter* than to *Salmonella* ratios? The total estimated cases of these illnesses would increase by a factor of 40. The inadequacy of simply applying a *Salmonella*-based multiplier to the number of cases reported from outbreaks can be demonstrated by applying that multiplier to the total number of cases reported in all foodborne disease outbreaks, typically 15,000 to 20,000 per year (3,4). On the basis of these estimates, the number of foodborne illnesses would range from 5.7 million to 7.6 million, including illnesses caused by unknown agents.

The authors make similar assumptions for hospitalizations and deaths: unknown agents are estimated to account for 81% of hospitalizations and 65% of deaths due to foodborne illnesses. In a retrospective review of death certificate data similar to that used by Mead and colleagues, Perkins et al. projected the number of unexplained deaths possibly due to infectious diseases they expected to find in the Emerging Infections Program sites (5). Prospectively, a much smaller number of unexplained deaths was actually found, because known causes were identified through a detailed review of the death certificates and cases (6). A prospective examination of death certificates for foodborne diseases might also result in a smaller than expected yield.

The need to rely on assumptions to generate estimates highlights the gaps in our understanding of foodborne diseases. A dozen different studies could address these data gaps. However, once the 76 million figure is agreed upon, the perceived need for these studies will decrease.

Finally, if these estimates are accepted as reasonable, do current food safety efforts represent sound public policy? If 82% of foodborne illnesses, 81% of hospitalizations, and 65% of deaths are caused by agents we have not yet identified, where is the commitment of resources needed to identify them? If eradicating *Campylobacter*, *Salmonella, Escherichia coli* O157:H7, and *Listeria* would reduce the number of foodborne illnesses by only 5%, hospitalizations by 10%, and deaths by 25%, why are these agents the primary focus of our national foodborne disease control efforts? Overestimating the occurrence of foodborne diseases caused by unknown agents may lead us to undervalue the public health importance of these and other well-known agents.

Estimating the occurrence of foodborne diseases is daunting. The numerous efforts, including this one by Mead et al., to provide estimates have serious shortcomings. The real challenge is to identify the gaps in our knowledge so that they can be systematically addressed and updated estimates of foodborne illness can be provided to guide prevention efforts and assess the effectiveness of current food safety measures (2).

**Craig Hedberg**
University of Minnesota,
Minneapolis, Minnesota, USA

### References

1. Mead PS, Slutsker L, Dietz V, McCaig LF, Bresee JS, Shapiro C, et al. Food-related illness and death in the United States. Emerg Infect Dis 1999;5:607-25.
2. Centers for Disease Control and Prevention. CDC data provides the most complete estimate on foodborne disease in the United States. Press release available at URL: http://www.cdc.gov/od/oc/media/pressrel/r990917.htm
3. Foodborne disease outbreaks, 5-year summary, 1983-1987. MMWR Morb Mortal Wkly Rep 1992;39(SS-1):1-15.
4. Surveillance for foodborne disease outbreaks. United States, 1988-92. MMWR Morb Mortal Wkly Rep 1996;45(SS-5):2-55.
5. Perkins BA, Flood JM, Danila R, Holman RC, Reingold AL, Klug LA, et al. Unexplained deaths due to possibly infectious causes in the United States: defining the problem and designing surveillance and laboratory approaches. Emerg Infect Dis 1996;2:47-53.
6. Minnesota Department of Health. Annual summary of communicable diseases reported to the Minnesota Department of Health, 1998. Disease Control Newsletter 1999;27:29-30.

### Food-Related Illness and Death in the United States–Reply to Dr. Hedberg

**To the Editor:** Like all scientific undertakings, our estimates require assumptions. Because the actual frequency of foodborne transmission of unknown agents cannot be measured directly, it must be assumed. If unknown agents had transmission characteristics similar to those of rotavirus (1% foodborne transmission) or cryptosporidium (10% foodborne transmission), as Dr. Hedberg suggests, the number of cases of foodborne illness caused by unknown agents would be substantially lower than we estimated. However, unknown agents could just as easily have the transmission characteristics of *Escherichia coli* O157:H7 or *Campylobacter* (80% foodborne transmission), which just 30 years ago were “unknown agents.” For the sake of objectivity, we based our assumption on the aggregate of information for known pathogens rather than on “expert opinion.” Interestingly, however, the Council of Science and Technology’s “expert opinion” of the percentage of diarrheal illness due to foodborne transmission was 35% (1), nearly identical to the figure we developed.

As noted in our article, pathogen-specific multipliers for underreporting are needed for many diseases. For lack of a better model, we assumed that the underreporting of toxin-mediated diseases follows the model of *Salmonella*. The alternative Dr. Hedberg suggests, *Campylobacter*, is also a nontoxin-mediated bacterial infection like *Salmonella*, but one for which the degree of underreporting is less well documented. Extrapolating from outbreak data to the number of sporadic cases does indeed have limitations, which is the reason we used it for only the few diseases for which other surveillance data were not available.

Regarding deaths attributed to unknown agents, prospective studies may show that some of these deaths are in fact caused by known agents. However, this would not necessarily lessen the overall impact of foodborne illness: it would merely shift the number of deaths from the unknown category to the known category. The possibility that some deaths attributed to unknown agents are in fact caused by *Salmonella* and other known pathogens supports our use of data on known pathogens to estimate the frequency of foodborne transmission for unknown agents.

Improved estimates will require expanded research into the etiologic spectrum of undiagnosed illness. In the meantime, documenting the substantial impact of foodborne illness neither devalues current surveillance and prevention efforts nor undermines future efforts to determine the causes and impact of foodborne diseases. Our estimates help define gaps in existing knowledge and provide a more rational basis for public health policy than reliance on decades-old data.

**Paul S. Mead, Laurence Slutsker, Patricia M. Griffin, Robert V. Tauxe**
Centers for Disease Control and Prevention, Atlanta, Georgia, USA

### References

1. Foodborne pathogens: risks and consequences. Ames, (IA): Council of Agricultural Science and Technology; 1994.

### Specimen Collection for Electron Microscopy

**To the Editor:** We read with interest the excellent article “Smallpox: an attack scenario,” by Tara O’Toole (1). At a critical point in the scenario, the author states, “The infectious disease specialist takes a swab specimen from the … skin lesions… and requests that it be examined by an experienced technician…. electron microscopy shows an orthopoxvirus consistent with variola.” In fact, swab specimens of skin lesions for the detection by electron microscopy of viruses such as pox and herpes viruses are far from ideal; the chances of viral detection would be greatly enhanced if a skin scraping were provided to the electron microscopist.

**J.A. Marshall and M.G. Catton**
Victorian Infectious Diseases Reference Laboratory, North Melbourne, Australia

### References

1. O’Toole T. Smallpox: an attack scenario. Emerg Infect Dis 1999;5:540-6.

**To the Editor:** Like all scientific undertakings, our estimates require assumptions. Because the actual frequency of foodborne transmission of unknown agents cannot be measured directly, it must be assumed. If unknown agents had transmission characteristics similar to those of rotavirus (1% foodborne transmission) or cryptosporidium (10% foodborne transmission), as Dr. Hedberg suggests, the number of cases of foodborne illness caused by unknown agents would be substantially lower than we estimated. However, unknown agents could just as easily have the transmission characteristics of *Escherichia coli* O157:H7 or *Campylobacter* (80% foodborne transmission), which just 30 years ago were "unknown agents." For the sake of objectivity, we based our assumption on the aggregate of information for known pathogens rather than on "expert opinion." Interestingly, however, the Council of Science and Technology's "expert opinion" of the percentage of diarrheal illness due to foodborne transmission was 35% (1), nearly identical to the figure we developed.

As noted in our article, pathogen-specific multipliers for underreporting are needed for many diseases. For lack of a better model, we assumed that the underreporting of toxin-mediated diseases follows the model of *Salmonella*. The alternative Dr. Hedberg suggests, *Campylobacter*, is also a nontoxin-mediated bacterial infection like *Salmonella*, but one for which the degree of underreporting is less well documented. Extrapolating from outbreak data to the number of sporadic cases does indeed have limitations, which is the reason we used it for only the few diseases for which other surveillance data were not available.

Regarding deaths attributed to unknown agents, prospective studies may show that some of these deaths are in fact caused by known agents. However, this would not necessarily lessen the overall impact of foodborne illness: it would merely shift the number of deaths from the unknown category to the known category. The possibility that some deaths attributed to unknown agents are in fact caused by *Salmonella* and other known pathogens supports our use of data on known pathogens to estimate the frequency of foodborne transmission for unknown agents.

Improved estimates will require expanded research into the etiologic spectrum of undiagnosed illness. In the meantime, documenting the substantial impact of foodborne illness neither devalues current surveillance and prevention efforts nor undermines future efforts to determine the causes and impact of foodborne diseases. Our estimates help define gaps in existing knowledge and provide a more rational basis for public health policy than reliance on decades-old data.
